# Supplementary material for: Using Community Science to Reveal the Global Chemogeography of River Metabolomes
Source: Metabolites. 2020 Dec 20;10(12):518. doi: 10.3390/metabo10120518 (PMC7767024; doi:10.3390/metabo10120518)
Supplement: Supplementary file 1 [file metabolites-10-00518-s001.zip › Supplementary_Information_12-14-2020/Supplemenatry_File_1_US_Maps_Properties_El_groups_Classes/US_Maps_Class_Cond Hydrocarbon.pdf]

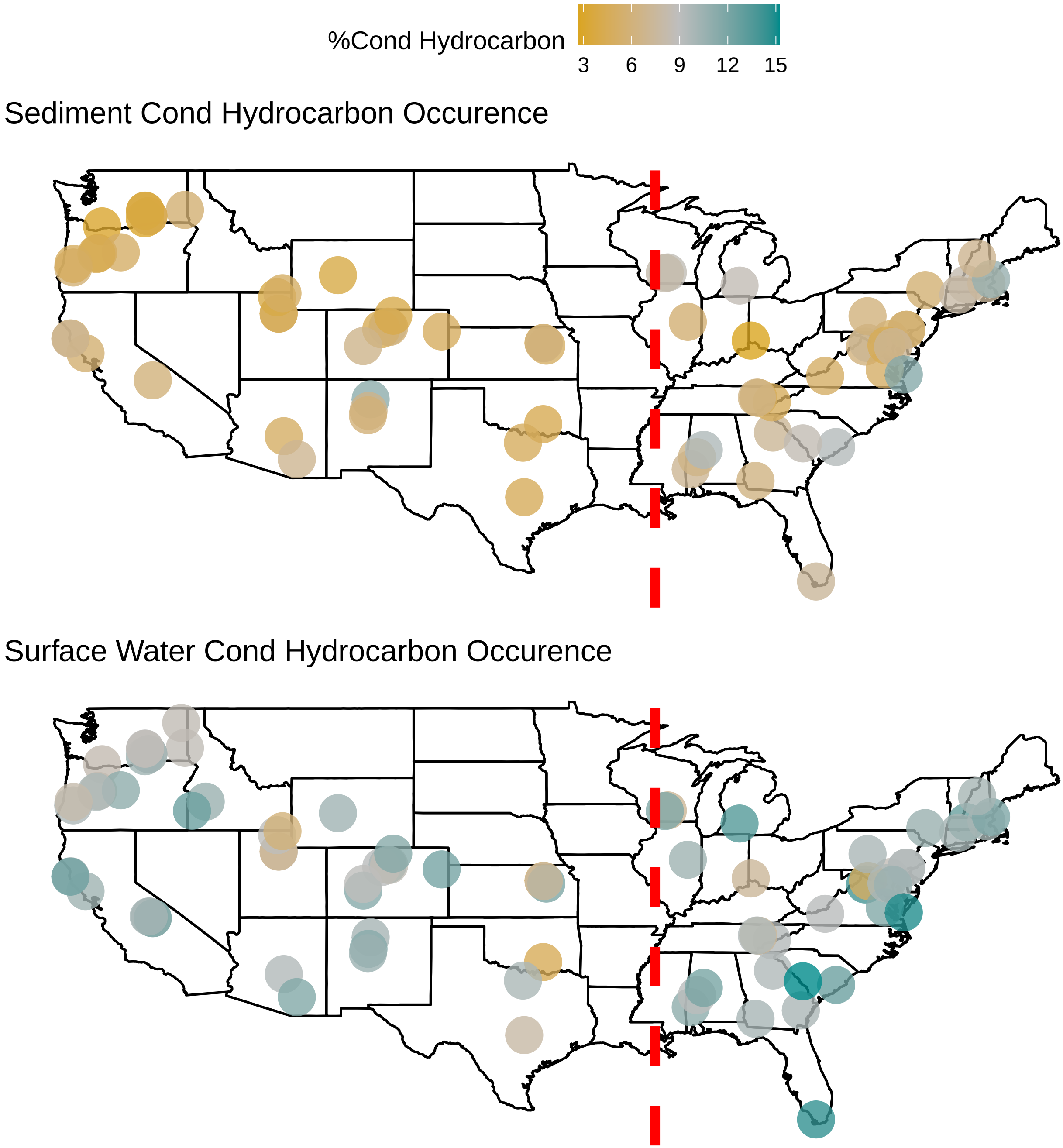

%Cond Hydrocarbon
3
6
9
12
15
Sediment Cond Hydrocarbon Occurence
Surface Water Cond Hydrocarbon Occurence
