# Supplementary material for: Using Community Science to Reveal the Global Chemogeography of River Metabolomes
Source: Metabolites. 2020 Dec 20;10(12):518. doi: 10.3390/metabo10120518 (PMC7767024; doi:10.3390/metabo10120518)
Supplement: Supplementary file 1 [file metabolites-10-00518-s001.zip › Supplementary_Information_12-14-2020/Supplementary_Figure_1_PCA_with_all_peaks.pdf]

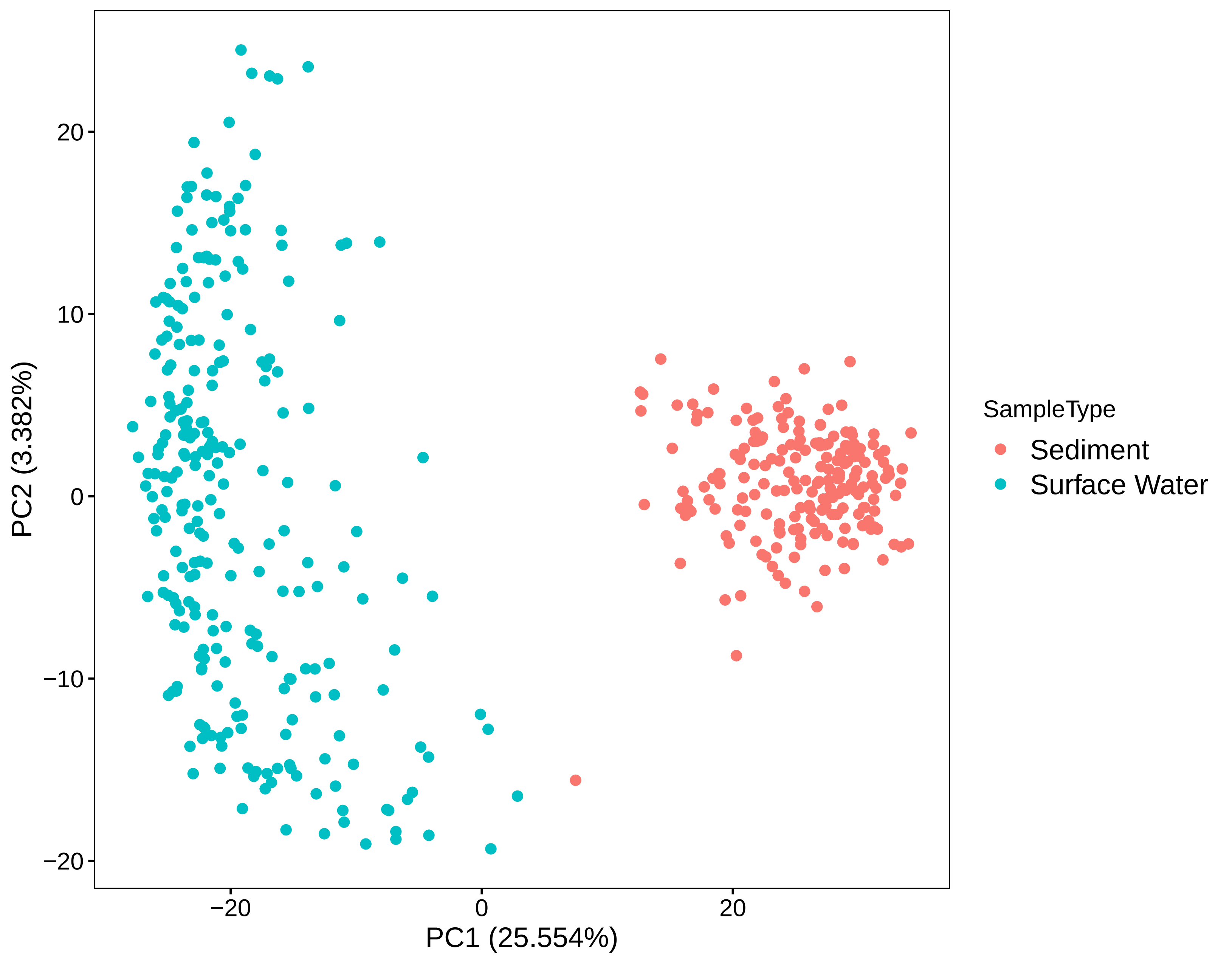

PC2 (3.382%)
20
10
0
−10
−20
−20
0
20
PC1 (25.554%)
SampleType
Sediment
Surface Water
